# NXT007-mediated hemostatic potential is suppressed by activated protein C-catalyzed inactivation of activated factor V

**DOI:** 10.1016/j.rpth.2023.102271

**Published:** 2023-11-23

**Authors:** Yuto Nakajima, Kenichi Ogiwara, Keito Inaba, Takehisa Kitazawa, Keiji Nogami

**Affiliations:** 1 Department of Pediatrics, Nara Medical University, Kashihara, Nara, Japan; 2 Advanced Medical Science of Thrombosis and Hemostasis, Nara Medical University, Kashihara, Nara, Japan; 3 Research Division, Chugai Pharmaceutical Co, Ltd, Yokohama, Japan

**Keywords:** antibodies, anticoagulants, factor V, factor VIII, hemophilia A

## Abstract

**Background:**

Activated protein C (APC) inactivates activated factor (F) V (FVa) and FVIIIa. NXT007, an emicizumab-based engineered therapeutic bispecific antibody, enhances the coagulation potential of FVIII-deficient plasma (FVIIIdef-plasma) to near normal levels. However, little is known about the effect of APC-induced inactivation in NXT007-mediated hemostatic function.

**Objectives:**

To investigate the contribution of APC-mediated reactions to NXT007-driven hemostasis.

**Methods:**

In pooled normal plasma (PNP) or FVIIIdef-plasma spiked with NXT007 (10 μg/mL), effects of APC (0-16 nM) were measured using a thrombin generation assay (TGA). The direct effects of APC on cofactor activity of NXT007 or FVIIIa in a FXa generation assay were also measured. The FVdef-plasma and FV Leiden plasma (FV_Leiden_ plasma) were preincubated with 2 anti-FVIII monoclonal antibodies (termed FVIII-depleted), and the APC effect in the presence of NXT007 in FVIII-depleted FVdef-plasma with the addition of exogenous FV (7.5-30 nM) or FVIII-depleted FV_Leiden_ plasma was investigated.

**Results:**

The APC dose-dependent suppression effect in TGA of FVIIIdef-plasma spiked with NXT007 was similar to that of PNP. FXa generation with NXT007 was not impaired by the addition of APC, suggesting that the APC-induced reaction in TGA with NXT007 was attributed to the direct inactivation of FVa. The addition of APC to FVIII-depleted FVdef-plasma, along with NXT007 and various FV concentrations, showed a similar attenuation to PNP. The NXT007-driven thrombin generation in FVIII-depleted FV_Leiden_ plasma was suppressed by APC, similar to the reaction in native FV_Leiden_ plasma.

**Conclusion:**

NXT007 did not impair APC-mediated downregulation of FVa in FVIIIdef-plasmas, regardless of the presence of FV mutation with APC resistance.

## Essentials

• NXT007 raises the coagulation function in patients with hemophilia A (PwHA) to a normal level.
• The activated protein C (APC)-mediated regulation of NXT007-driven hemostasis in PwHA remains to be examined.
• APC suppressed the coagulation potential in PwHA with NXT007 and in normal plasma.
• APC can regulate the coagulation function of PwHA with NXT007 through factor Va inactivation.

## 1 Introduction

Emicizumab (Hemlibra) is a recombinant, humanized, therapeutic bispecific monoclonal antibody (mAb) that binds to factor (F) IX/activated FIX (FIXa) and FX/FXa and mimics FVIIIa cofactor function in the intrinsic tenase complex [1,2]. Emicizumab prophylaxis significantly reduces bleeding episodes in patients with severe hemophilia A (HA), regardless of the presence of inhibitors [[3], [4], [5], [6], [7], [8], [9]]. On the other hand, the concomitant treatment with bypassing agents or FVIII products is still required for managing severe breakthrough bleeds or major surgeries in some cases of emicizumab-treated patients with HA assumedly because *in vitro* coagulation potentials and bleeding patterns at a clinically therapeutic emicizumab concentrations appeared to be similar to those at mild HA [10]. Therefore, the improved bispecific antibody mimicking a FVIIIa function possessing higher cofactor activity than emicizumab should be further beneficial for these patients.

In this context, an emicizumab-based, engineered, therapeutic bispecific antibody (termed NXT007) has been created as the next-generation treatment with more potent activity [11]. The addition of NXT007 in plasmas of patients with HA showed a coagulation potential up to approximately 100 IU/dL of FVIII when evaluated by a tissue factor (TF)-triggered thrombin generation assay (TGA) [11]. The FVIIIa-mimicking coagulation potential of NXT007 was significantly greater than emicizumab because the *K*_d_ value of NXT007 to FX/FXa was 30-40-fold lower than that of emicizumab [11]. The clinical trial (phase 1 NXT007_Hemophilia-A_NXT001JG) of this product for patients with HA is currently ongoing in the East Asian region (JapicCTI-194919).

The coagulation system and the anticoagulant system maintain a good balance for physiological hemostasis. Protein C (PC) is known as a key component of the anticoagulant factor, and activated PC (APC) plays a crucial role in natural anticoagulant mechanisms [12]. The potential development of thrombosis is well controlled by APC and protein S (PS). APC and PS diminish the activity of FVa and FVIIIa [13] and inactivate FVa by cleaving at Arg306, Arg506, and Arg679 [14]. Arg506 cleavage is kinetically favored over Arg306 cleavage but only partially regulates FVa by inhibiting FXa bound to FVa [15]. Cleavage at both Arg506 and Arg306 results in near complete loss of FVa activity [14]. With regard to FVIII/FVIIIa, APC and PS regulate FVIIIa following cleavage of FVIIIa at residues Arg336 and Arg562 [16]. However, the extent to which APC contributes to the inactivation of FVa and FVIIIa remains to be fully understood.

APC resistance (APCR) is known as a major risk factor for venous thromboembolism [17,18]. FV-Arg506Gln (FV Leiden; FV_Leiden_) exhibits APCR because APC fails to cleave Arg506 efficiently, resulting in thrombophilia [17,18]. In addition to FV_Leiden_-dependent APCR, APCR, due to other causes, also increases the risk of venous thrombosis [17], indicating the clinical impact of the APC-dependent inactivation pathway. We previously demonstrated that emicizumab-driven hemostasis was downregulated by APC-mediated FVa inactivation [19]. However, the role of APC-dependent suppression in NXT007-mediated hemostasis remains to be investigated.

In our previous study, we demonstrated that ellagic acid (Elg)/TF-triggered TGA is useful for evaluating APC-mediated inactivation in emicizumab-driven hemostasis [19]. The reason for using an Elg/TF-triggering condition was to overcome critical assay limitations in the other triggering conditions: the enhancing effects of FVIIIa-function mimetic bispecific antibodies appeared too rapidly when using contact activator (Elg)-triggered TGA and were not sufficiently sensitive when using TF-triggered TGA [19]. In addition, we advocated in another study that Elg/TF-triggered TGA could evaluate global coagulation potential during emicizumab prophylaxis, even in the concomitant therapy with FVIII or bypassing agents [20]. It may mean that the Elg/TF-triggering condition could reflect the coagulation reactions in the presence of a FVIIIa-function mimetic bispecific antibody more comprehensively than the pure extrinsic or intrinsic triggering condition. In the present study, we examined the regulatory effects of APC in NXT007-driven hemostasis using a FXa generation assay and Elg/TF-triggered TGA.

## 2 Methods

This study was approved by the Medical Research Ethics Committee of Nara Medical University (No. 2712).

### 2.1 Reagents

Recombinant emicizumab and NXT007 were produced using a Chinese hamster ovary cell line [6,16]. The recombinant FVIII preparation (Advate; Takeda), FV- and FVIII-deficient plasma (George King), FV_Leiden_ plasma (Trina Bioreactive), plasma-derived (pd-)APC, PS, FV (Haematologic Technologies Inc), recombinant hirudin (Calbiochem), recombinant human TF (Innovin; Dade), Elg (Sysmex), activated partial thromboplastin time (aPTT) reagent (Thrombocheck APTT-SLA; Sysmex Corporation), prothrombin time (PT) reagent (Revohem PT; Sysmex Corporation), a thrombin-specific fluorogenic substrate (Z-Gly-Gly-Arg-AMC, Bachem), and FXa substrate S-2222 (Chromogenix) were purchased from the indicated vendors. Phospholipid (PL) vesicles (phosphatidylserine:phosphatidylcholine:phosphatidylethanolamine = 1:6:3) were prepared using *N*-octylglucoside [21]. An anti-A2 mAb (VIII-2236) and an anti-C2 mAb (VIII-9222) were provided by Chugai Pharmaceutical Co, Ltd [22].

### 2.2 Plasma samples

Pooled normal plasma (PNP) was prepared from 20 normal healthy individuals. Whole blood samples were collected in plastic tubes containing 3.2% sodium citrate at a ratio of 9:1 (Fuso Pharmaceutical Industries). No study subjects had taken any medication that may have influenced platelet or coagulation function 2 weeks prior to blood sampling. Platelet-poor plasma was separated by centrifuging citrated whole blood for 10 minutes at 2000 × g. All plasma samples were stored at −80 °C and thawed at 37 °C immediately prior to the assays.

### 2.3 Preparation of plasmas containing NXT007

NXT007 at the indicated concentrations was incubated for 30 minutes at 37 °C with FVIII-deficient plasma, and PNP or FV-deficient plasma preincubated with 2 anti-FVIII mAbs at a final concentration (f.c.) of 16 BU/mL (termed FVIII-depleted plasma). We confirmed that FVIII:C is undetectable in FVIII-depleted plasma by both the one-stage clotting assay and clot waveform analysis (CWA) as previously established [23].

### 2.4 Thrombin generation assay

TGA was performed as previously described [20]. Briefly, plasma samples (80 μL) were preincubated for 10 minutes with 20 μL of a trigger reagent containing TF, Elg, and PL (f.c. 0.5 pM, 0.3 μM, and 4 μM, respectively). After adding 20 μL of a reagent containing CaCl_2_ and fluorogenic substrate (f.c. 16.7 mM and 2.5 mM, respectively), the development of fluorescent signals was monitored using a Fluoroskan Ascent microplate reader (Thermo Fisher Scientific). Data analyses were performed using the manufacturer's software to derive the following standard parameters: lag time, time to the peak, peak thrombin (PeakTh), and endogenous thrombin potential (ETP). The PeakTh inhibition was calculated as follows: ([PeakTh in PNP or NXT007 without APC − PeakTh in PNP or NXT007 with APC]/[PeakTh in PNP or NXT007 without APC]) × 100.

### 2.5 PT/activated partial thromboplastin time-triggered modified clot waveform analysis

Modified CWA was performed using a CS-2400i instrument (Sysmex) with a trigger mixture comprising TF and Elg [20]. Briefly, plasma samples (50 μL) were preincubated for 3 minutes with PT and aPTT trigger reagents (50 μL; PT/aPTT/buffer = 1/15/135 [v/v/v]) prior to the addition of 25 mM CaCl_2_ (50 μL) to initiate coagulation. The automated coagulation analyzer detected the transmittance of light intensity, and clot waveforms were computer-processed by the commercial kinetic algorithm. The |min1| was calculated as the minimum value of the first derivatives of the transmittances, reflecting the maximum coagulation velocity achieved. The minimum transmittance (0%) was determined at the immediate postcoagulation phase, and the adjusted-|min1| (Ad|min1|) was defined as the maximum coagulation velocity obtained from the first derivatives of adjusted waveform [20]. The Ad|min1| value and clot time obtained from healthy individuals were 7.2 ± 0.6 and 31 ± 1.6 (mean ± SD).

### 2.6 Factor VIIIa degradation assay

The rate of FX conversion to FXa was monitored using a purified system [19]. FVIII (2 nM) in buffer (20 mM HEPES, 0.1 M NaCl, 5 mM CaCl_2_, pH 7.2, 0.01% Tween 20) containing PL vesicles (20 μM) was activated by the addition of thrombin (5 nM). Thrombin activity was terminated after 30 seconds by the addition of hirudin (2.5 U/mL). The FVIIIa or NXT007 (10 μg/mL) was then incubated for 20 minutes with APC at various concentrations (0-2 nM), PS (5 nM), and FV (1 nM). FXa generation in aliquots of the reaction mixtures was initiated by the addition of FIXa (5 nM) and FX (200 nM). The reactions were quenched after 1 minute by adding 50 mM ethylene-diamine-tetraacetic acid. The amount of FXa generated was determined by the addition of the chromogenic substrate S-2222 (f.c. 0.46 mM), and the velocity rates of FXa generation were calculated. All reactions were performed at 23 °C.

### 2.7 Statistical analysis

Data are presented as the average and SD. Data analysis was performed using KaleidaGraph (Synergy). Significant differences were determined by Dunnett’s multiple comparison test. *P* values < .05 were considered statistically significant.

## 3 Results

### 3.1 Coagulation potential in factor VIII-deficient plasma spiked with NXT007 or emicizumab

**Figure 1:**
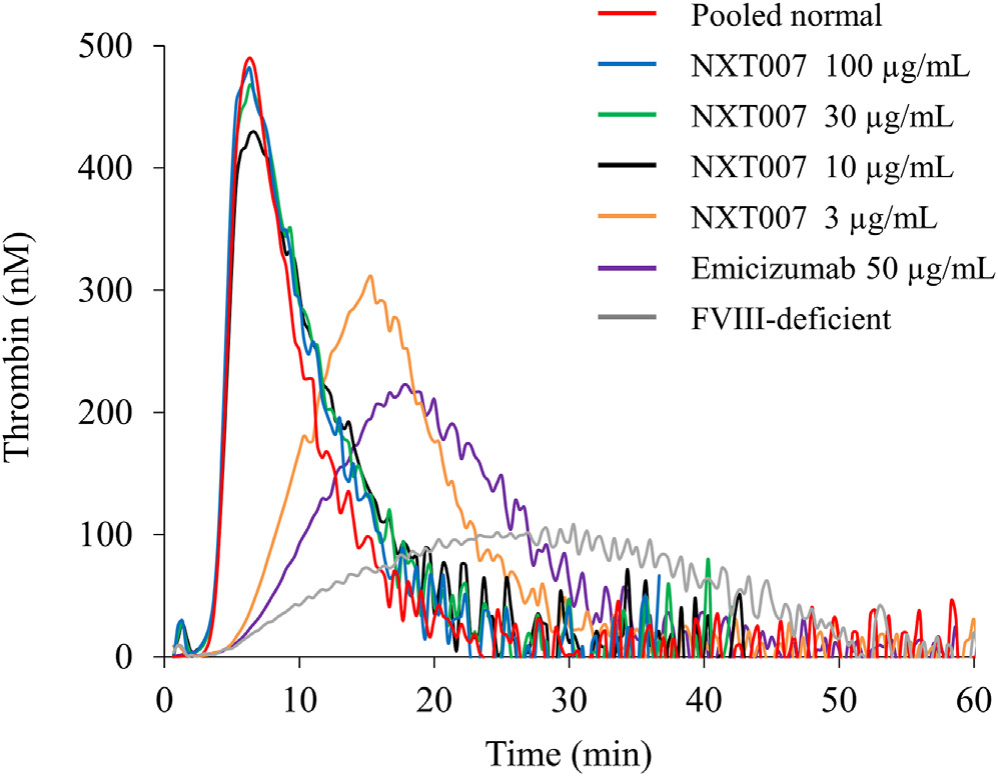
Thrombin generation potentials in factor VIII (FVIII)-deficient plasmas spiked with emicizumab or NXT007. Emicizumab (50 μg/mL) or NXT007 (3-100 μg/mL) were added to FVIII-deficient plasma. These samples were incubated with tissue factor (1 pM), ellagic acid (0.3 μM), and phospholipid (PL) vesicles (4 μM) prior to the addition of fluorogenic substrate and CaCl_2_ at the start of the assay as described in the Methods section. Experiments were performed three times, and representative thrombin generation curves are shown (red: pooled normal plasma; blue: NXT007 100 μg/mL; green: NXT007 30 μg/mL; black: NXT007 10 μg/mL; orange: NXT007 3 μg/mL; purple: emicizumab 50 μg/mL; gray: FVIII-deficient plasma).

To examine the coagulation potential of NXT007, NXT007 (3-100 μg/mL) or emicizumab (50 μg/mL; clinically therapeutic concentration) was added to FVIII-deficient plasma, followed by measuring the coagulation potential using TGA. Representative curves are shown in Figure 1, and the parameters obtained from the samples are summarized in Table 1. As expected, the PeakTh of FVIII-deficient plasma in the presence of emicizumab was significantly greater than that of FVIII-deficient plasma (*P* < .01). The PeakTh of FVIII-deficient plasma supplemented with NXT007 (3 μg/mL) was significantly greater than that supplemented with emicizumab (*P* < .01). The PeakTh level of FVIII-deficient plasma in the presence of NXT007 (10, 30, and 100 μg/mL) was significantly higher than that in the presence of emicizumab (*P* < .01), and the PeakTh level in NXT007 at 10, 30, and 100 μg/mL was similar to that in PNP (*P* > .05). On the other hand, the ETPs in FVIII-deficient plasma with NXT007 (3-100 μg/mL) were similar to that with emicizumab. Since the ETP did not change in a NXT007 dose-dependent manner, this parameter appeared unlikely to be useful for reflecting coagulation function as previously described [24]. Overall, these results demonstrate that the NXT007 could restore the coagulant potential to near-normal level.

**Table 1:** Parameters in factor VIII-deficient plasma supplemented with NXT007 or emicizumab obtained by thrombin generation assay.

	Lag time *min*	Peak thrombin *nM*	ETP *nM × min*
Emicizumab, 50 μg/mL	6.3 ± 1.1	213 ± 27	3290 ± 830
NXT007, 3 μg/mL	5.9 ± 0.9	333 ± 66^a^	3769 ± 518
NXT007, 10 μg/mL	4.9 ± 1.1	404 ± 27^a^	3688 ± 510
NXT007, 30 μg/mL	4.9 ± 1.1	454 ± 44^a^	3867 ± 451
NXT007, 100 μg/mL	5.1 ± 1.3	447 ± 29^a^	3802 ± 337
FVIII-deficient plasma	6.7 ± 0.6	101 ± 3^a^	2843 ± 256
Pooled normal plasma	5.4 ± 0.3	470 ± 13^a^	3739 ± 307

Thrombin generation assay (TGA) was performed as described in the Methods section. TGA parameters of emicizumab, NXT007, and factor VIII-deficient or pooled normal plasma are shown. TGA parameters in emicizumab were used as the control group, and those between emicizumab and NXT007 or FVIII-deficient plasma or pooled normal plasma were compared. Significant differences were considered when *P* < .05. Experiments were performed 3 times, and the average values and SD are shown.

ETP, endogenous thrombin potential; FVIII, factor VIII.

a *P* < .01 vs emicizumab 50 μg/mL.

### 3.2 Impacts of activated protein C-mediated downregulation in the plasma of patients with hemophilia A spiked with NXT007

**Figure 2:**
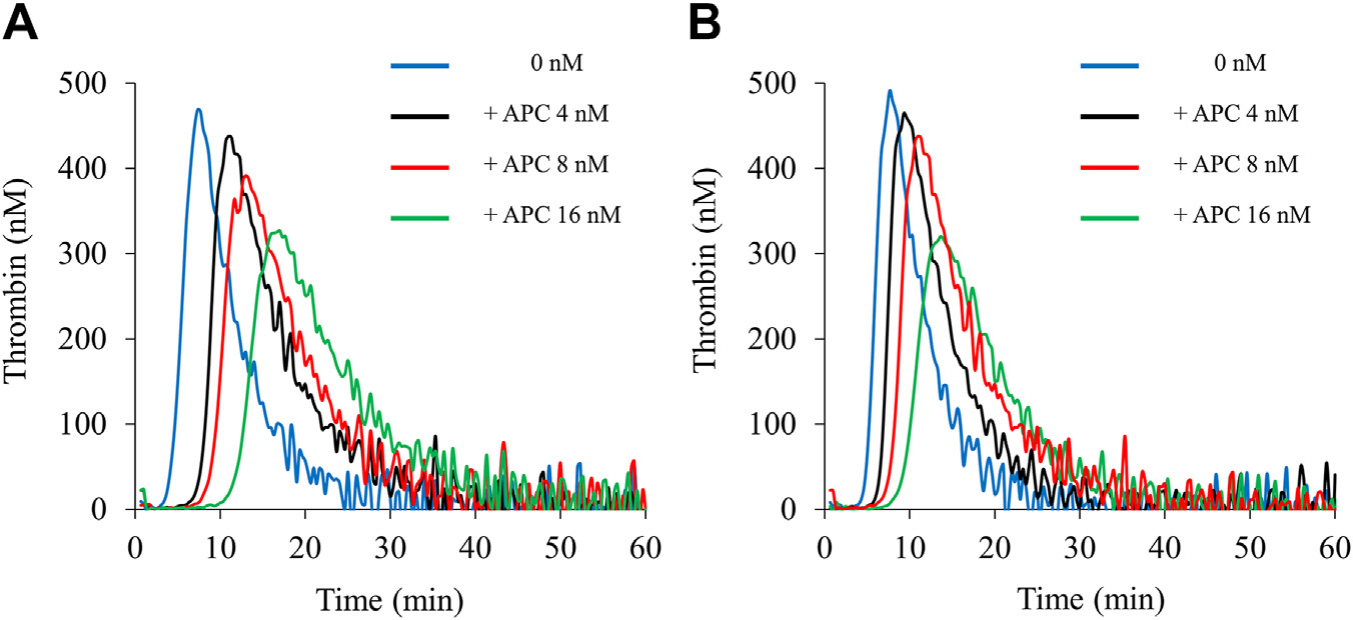
Effect of activated protein C (APC) in normal plasma or factor VIII-deficient plasmas spiked with NXT007 by thrombin generation assay. Tissue factor/ellagic acid-triggered thrombin generation after the addition of various APC concentrations (0-16 nM) was examined in FVIII-deficient plasma supplemented with NXT007 (10 μg/mL) (A) or normal plasma (B) as described in Methods section. Experiments were performed 3 times, and representative APC dose-dependent thrombin generation curves are shown (blue: no addition; black: APC 4 nM; red: APC 8 nM; green: APC 16 nM).

In the previous study, the *in vitro* addition of NXT007 at 10 μg/mL exhibited a higher thrombin generation activity in FVIII-deficient plasma than that of recombinant human FVIII at 40 IU/dL (the lower limit of the normal range) without prolonged prothrombin time of the plasma [11]. Based on a nonclinical dataset, 10 μg/mL was expected to be within the target plasma levels of NXT007 in clinical settings. Indeed, our results in Figure 1 demonstrate that the *in vitro* addition of NXT007 at 10 μg/mL restored PeakTh closely to that in PNP. Therefore, we set the concentration of NXT007 at 10 μg/mL and continued the experiments thereafter. To examine the impact of APC-mediated downregulation on the hemostatic reactions mediated by NXT007, TGA in FVIII-deficient plasmas spiked with NXT007 (the NXT007-mediated condition) was performed in the presence of various concentrations of APC (0-16 nM). We also evaluated the effects of APC (0-16 nM) on TGA in PNP, which was mediated by FVIII, for comparison. Representative curves are illustrated in Figure 2. The TGA parameters obtained from the measurements are summarized in Table 2. The addition of APC (16 nM) to the NXT007-mediated condition decreased PeakTh from 449 ± 15 to 330 ± 3 nM (*P* < .01), with a 27% inhibition of PeakTh. Also, in PNP, the addition of APC reduced PeakTh from 485 ± 13 to 349 ± 13 nM (*P* < .01), with a 28% inhibition of PeakTh. The percentage inhibition of PeakTh mediated by NXT007 under APC at 4 and 8 nM was 5% and 16%, respectively, while the percentage inhibition of PeakTh in PNP under APC at 4 and 8 nM was 5% and 10%, respectively. Thus, the inhibitory effect of APC on PeakTh in the NXT007-mediated coagulation was found to be similar to that in PNP. Furthermore, APC prolonged lag time both in PNP and the NXT007-mediated condition. In contrast, ETP was not significantly changed by the addition of APC both in PNP and the NXT007-mediated condition. These results indicate that the TGA parameters, lag time, and PeakTh could be useful parameters for reflecting APC-catalyzed inactivation and that the effect of APC-mediated downregulation worked well in the NXT007-mediated condition in a similar manner to PNP.

**Table 2:** Parameters in normal plasma or factor VIII-deficient plasma with NXT007 in the presence of activated protein C by thrombin generation assay.

	Lag time *min*	Peak thrombin *nM*	ETP *nM × min*		Lag time *min*	Peak thrombin *nM*	ETP *nM × min*
PNP	4.1 ± 0.7	485 ± 13	3919 ± 788	NXT007	5.8 ± 1.2	449 ± 15	4284 ± 857
PNP + APC 4 nM	5.0 ± 1.0	460 ± 12	3420 ± 598	NXT007 + APC 4 nM	7.1 ± 1.6	415 ± 12	4095 ± 752
PNP + APC 8 nM	6.1 ± 1.4	438 ± 9^a^	3809 ± 57	NXT007 + APC 8 nM	8.0 ± 2.0	377 ± 27^b^	4022 ± 668
PNP + APC 16 nM	8.0 ± 1.4b	349 ± 13^b^	3929 ± 35	NXT007 + APC 16 nM	11.1 ± 3.0^a^	330 ± 3^b^	3758 ± 101

The thrombin generation assay (TGA) was performed as described in the Methods section. The obtained parameters in PNP or factor VIII-deficient plasma with NXT007 (10 μg/mL) together with various concentrations of APC are shown. TGA parameters in PNP or FVIII-deficient plasma plus NXT007 with or without APC were compared. Significant differences were considered when *P* < .05. Experiments were performed 3 times, and the average values and SD are shown.

APC, activated protein C; ETP, endogenous thrombin potential; PNP, pooled normal plasma.

a *P* < .05.

b *P* < .01 vs no APC.

### 3.3 Activated protein C does not contribute to downregulation of the NXT007-mediated factor Xa generation

**Figure 3:**
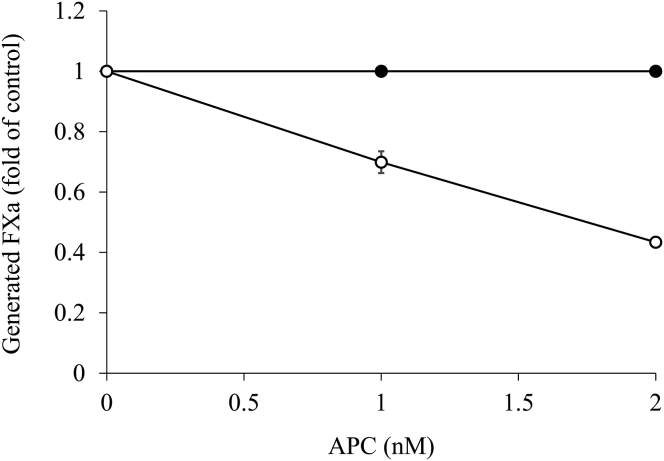
Effect of inactivation by activated protein C (APC) on NXT007 by factor Xa (FXa) generation assay. Factor VIII (2 nM) with phospholipid (20 μM) was incubated with thrombin (5 nM), followed by the addition of hirudin (2.5 U/mL). The generated FVIIIa (open circles) or NXT007 at 10 μg/mL (closed circles) was incubated with APC (0-2 nM) at 3 different concentrations, protein S (5 nM), and FV (1 nM) for 20 minutes. FXa generation was initiated by the addition of FIXa (5 nM) and FX (200 nM) for 1 minute, as described in Methods section. Generated FXa in the absence of APC was regarded as control. The average generated FXa with FVIIIa or NXT007 in the absence of APC was 83 or 32 nM/min, respectively, and these values were regarded as the initial levels. Experiments were performed 3 times, and the average values and SD are shown. Some SD bars are shorter than symbols.

We next proceeded with the investigation based on FXa generation to elucidate the mechanism of APC-mediated inactivation of hemostasis in FVIII-deficient plasmas spiked with NXT007 (the NXT007-mediated condition) as previously carried out for emicizumab [19]. To clarify the association of NXT007 with APC-catalyzed inhibition of FXa generation, we examined a FVIIIa degradation assay with NXT007 (10 μg/mL) or with FVIIIa (2 nM) after the addition of APC (0-2 nM), PS (5 nM), and FV (1 nM). FXa generation with FVIIIa was reduced by ∼60% at maximum level in an APC dose-dependent manner, whereas FXa generation with NXT007 was not impaired (Figure 3). These results indicated that NXT007, unlike FVIIIa, is not directly inactivated by APC and that inactivation of FVa by APC plays an important role in APC-mediated inactivation in the NXT007-mediated condition.

### 3.4 Impact of factor V/factor Va on activated protein C-mediated inactivation in the plasma of patients with hemophilia A spiked with NXT007

**Figure 4:**
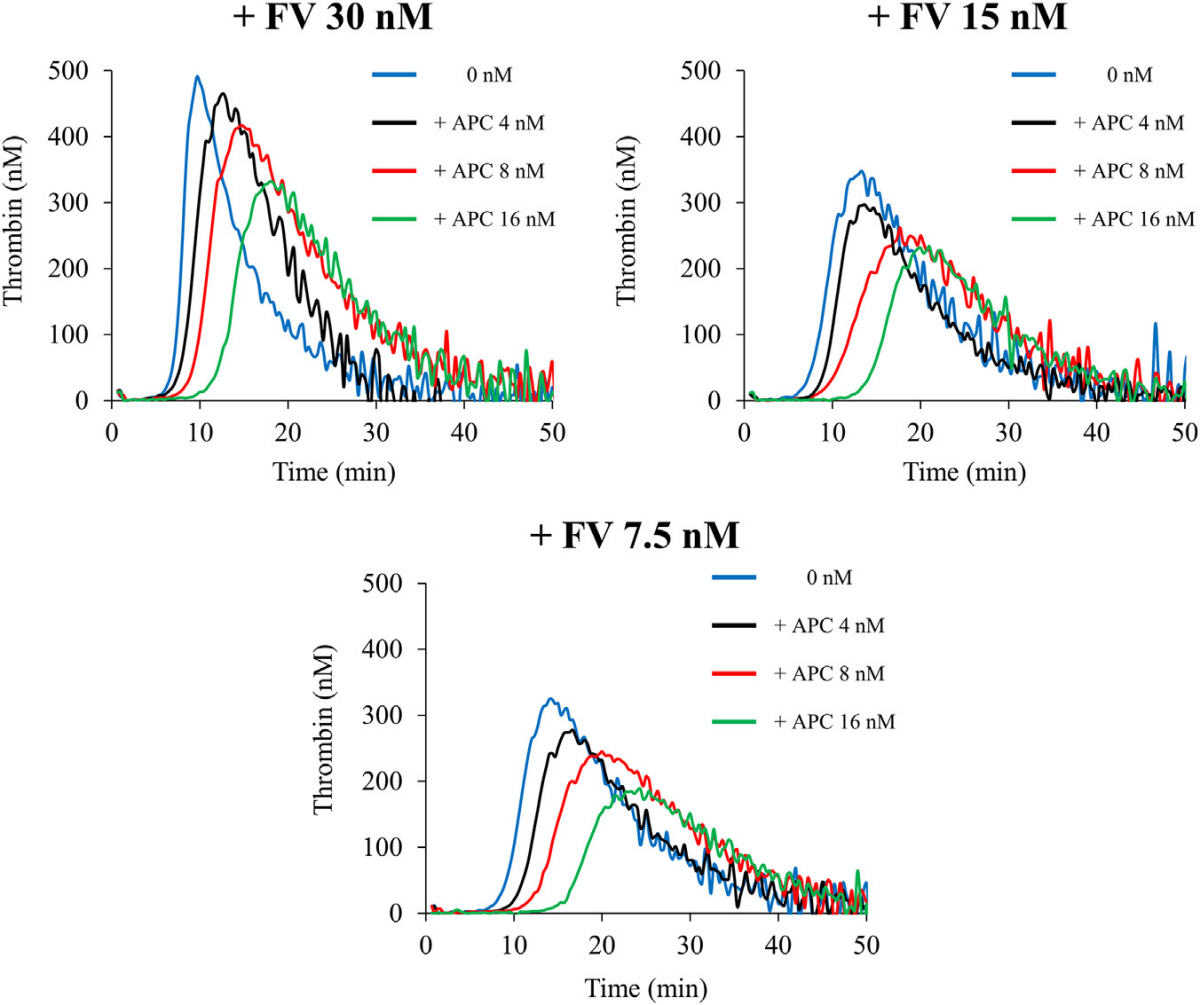
Impact of factor V(a) (FV) in activated protein C (APC)-induced downregulation on NXT007-driven thrombin generation. Thrombin generation in FV-deficient plasma preincubated with 2 anti-FVIII monoclonal antibodies together with exogenous FV at 30, 15, and 7.5 nM in the presence of NXT007 (10 μg/mL) was investigated as described in Methods section. Experiments were performed 3 times, and representative APC dose-dependent thrombin generation curves are shown (blue: no addition; black: APC 4 nM; red: APC 8 nM; green: APC 16 nM).

To elucidate the contribution of FV/FVa on APC-catalyzed inactivation in NXT007-mediated TG potential, FV-deficient plasma was preincubated with 2 anti-FVIII mAbs for preparing the FVIII-depleted FV-deficient plasmas. Then, the effect of APC on the TGA with NXT007 (10 μg/mL) and exogenous FV (7.5-30 nM; corresponding to 25%-100% of physiological concentration) in FVIII-depleted FV-deficient plasmas was assessed. Figure 4 illustrates that the PeakTh in the copresence of NXT007 and FV (7.5, 15, and 30 nM) without APC was 310 ± 12, 361 ± 39, and 444 ± 28 nM, respectively, and the PeakTh in the presence of NXT007 under low FV conditions (7.5 and 15 nM) was significantly lower than that under the physiological FV condition (30 nM). The condition in the presence of FV <3.7 nM could not be evaluated because of the lower TG potential in this condition (data not shown).

We next investigated the difference in APC-mediated downregulation in FVIII-depleted FV-deficient plasmas spiked with NXT007 (the NXT007-mediated condition) under all the FV concentrations employed (7.5, 15, and 30 nM). Under the presence of FV at 30 nM, the addition of APC (8 and 16 nM) to the NXT007-mediated condition significantly decreased PeakTh. Under the presence of FV at 7.5 or 15 nM, the addition of APC (8 and 16 nM) to the NXT007-mediated condition significantly decreased PeakTh (Table 3). Throughout all the FV concentrations used, percentage inhibition of PeakTh by APC at 8 and 16 nM in the NXT007-mediated condition ranged between 19% and 28% and 28% and 37%, respectively. As described above, in PNP, the percentage inhibition of PeakTh by APC at 8 and 16 nM was 10% and 28%, respectively. Therefore, APC-catalyzed FV(a) inactivation occurred similarly or slightly more intensely in the NXT007-mediated condition than in the FVIII-mediated condition (PNP) throughout all the FV concentrations employed. With regard to the other parameters in the NXT007-mediated condition, the addition of APC (16 nM) significantly prolonged lag time and time to the peak but did not affect ETP significantly (data not shown). Taken together, these results suggest that, although the hemostatic potential in the NXT007-mediated condition was affected by the concentration of FV, APC-catalyzed FV(a) inactivation facilitated the downregulation of NXT007-mediated hemostasis even under the low FV condition.

**Table 3:** Suppression effect by activated protein C in factor VIII-depleted factor V-deficient plasma supplemented with NXT007 in the presence of various factor V concentrations.

	Lag time *nM*	ttPeak *min*	Peak thrombin *nM*		Lag time *nM*	ttPeak *min*	Peak thrombin *nM*		Lag time *min*	ttPeak *min*	Peak thrombin *nM*
FV (7.5)	9.4 ± 0.5	15.9 ± 1.4	310 ± 12	FV (15)	7.8 ± 0.2	14.0 ± 1.4	361 ± 39	FV (30)	7.0 ± 0.3	11.2 ± 1.3	444 ± 28
FV (7.5) + APC (4)	11.3 ± 0.9	17.4 ± 1.1	280 ± 20	FV (15) + APC (4)	9.3 ± 0.2	15.6 ± 1.5	329 ± 49	FV (30) + APC (4)	8.2 ± 0.7	12.1 ± 0.3	429 ± 31
FV (7.5) + APC (8)	12.7 ± 1.6^a^	20.1 ± 2.6	239 ± 3^b^	FV (15) + APC (8)	11.8 ± 1.1^b^	18.3 ± 2.0	261 ± 14^a^	FV (30) + APC (8)	9.2 ± 1.0^a^	13.5 ± 1.3	361 ± 51^a^
FV (7.5) + APC (16)	17.6 ± 2.0^b^	23.3 ± 5.2^a^	196 ± 17^b^	FV (15) + APC (16)	14.3 ± 0.5^b^	21.3 ± 0.9^a^	242 ± 17^a^	FV (30) + APC (16)	11.1 ± 0.8^b^	15.7 ± 2.0^a^	319 ± 15^b^

The thrombin generation assay (TGA) was performed as described in the Methods section. The obtained parameters of factor VIII-depleted FV-deficient plasmas with NXT007 (10 μg/mL) in the presence of various FV conditions after the addition of APC are shown. TGA parameters in NXT007 in the presence of APC were compared with NXT007 in the absence of APC under each FV condition. Significant differences were considered when *P* < .05. Experiments were performed 3 times, and the average values and SD are shown.

APC (4), APC (8), APC (16), activated protein C 4, 8, 16 nM; FV (7.5), FV (15), FV (30), factor V 7.5, 15, 30 nM; ttPeak; time to the peak.

a *P* < .05.

b *P* < .01 vs no APC.

### 3.5 Activated protein C-catalyzed inactivation of NXT007 in factor VIII-depleted FV_Leiden_ plasma

**Figure 5:**
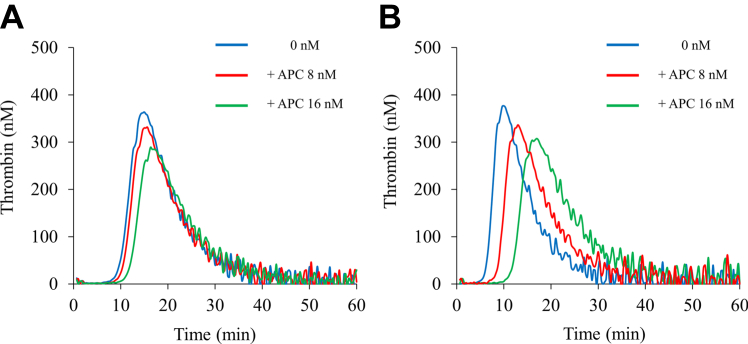
Activated protein C (APC)-mediated downregulation of thrombin generation in factor VIII-depleted FV Leiden (FV_Leiden_) plasma reacted with NXT007. Thrombin generation after the addition of various APC concentrations (0-16 nM) was performed in native FV_Leiden_ plasma (A) and FV_Leiden_ plasma preincubated with 2 anti-FVIII monoclonal antibodies in the presence of NXT007 (10 μg/mL) (B) as described in Methods section. Experiments were performed 2 times. The average peak thrombin of FV_Leiden_ plasma in the presence of APC at 0, 8, and 16 nM was 363 ± 6 nM, 332 ± 2 nM, and 283 ± 6 nM, respectively. The average peak thrombin of NXT007 in FVIII-depleted FV_Leiden_ plasma in the presence of APC at 0, 8, and 16 nM was 342 ± 50 nM, 300 ± 50 nM, and 265 ± 60 nM, respectively. The average lag time of FV_Leiden_ plasma in the presence of APC at 0, 8, and 16 nM was 8.7 ± 1.9 minutes, 10.1 ± 0.6 minutes, and 11.7 ± 0.6 minutes, respectively. The average lag time of NXT007 in FVIII-depleted FV_Leiden_ plasma in the presence of APC at 0, 8, and 16 nM was 8.9 ± 2.9 minutes, 11.3 ± 3.3 minutes, and 14.3 ± 3.3 minutes, respectively. Representative APC dose-dependent thrombin generation curves are shown (blue: no addition; red: APC 8 nM; green: APC 16 nM).

A previous study reported that 35 cases complicated with FV_Leiden_ mutation were detected among 700 subjects with hemophilia [25]. Therefore, we investigated whether NXT007 in FV_Leiden_ plasma could influence the effect of APC-catalyzed inactivation. We preincubated the FV_Leiden_ plasmas with 2 anti-FVIII mAbs for preparing FVIII-depleted FV_Leiden_ plasmas, and the impact of APC-mediated downregulation in native FV_Leiden_ plasmas and FVIII-depleted FV_Leiden_ plasmas supplemented with NXT007 (10 μg/mL) was investigated using TGA. Figure 5 shows that the average PeakTh of FV_Leiden_ plasma (363 ± 6 nM) was comparable to that of NXT007 in FVIII-depleted FV_Leiden_ plasma (342 ± 50 nM). The percentage inhibition of PeakTh by the addition of APC (16 nM) in FV_Leiden_ plasma was 22% (the FVIII-mediated condition). Also, in the NXT007-mediated condition, the percentage inhibition of PeakTh by the addition of APC (16 nM) in FVIII-depleted FV_Leiden_ plasma was 23%, indicating a similar inhibitory potential to FV_Leiden_ plasma alone (the FVIII-mediated condition). The average lag time of FV_Leiden_ plasma in the presence of APC at 16 nM was 1.3-fold longer than in the absence of APC (11.7 ± 0.7 minutes vs 8.7 ± 1.9 minutes, respectively). The lag time in FVIII-depleted FV_Leiden_ plasma spiked with NXT007 in the presence of APC at 16 nM was 1.6 times longer compared with that in the absence of APC (14.3 ± 3.3 minutes vs 8.9 ± 2.9 minutes, respectively), similar to native FV_Leiden_ plasma. These results suggest that NXT007-mediations did not disturb APC-catalyzed FVa inactivation even under the FV_Leiden_ condition.

## 4 Discussion

The APC anticoagulant pathway plays a crucial role in balancing the coagulation system. APCR has been found in 20% to 40% of patients with venous thrombosis [26,27], indicating a major role of APC in regulating coagulation. Our previous study indicated that APC could suppress emicizumab-mediated TG potential by inactivating FVa, even in the absence of FVIII [19]. NXT007 has a greater potential to promote coagulation in HA state than emicizumab [11]; therefore, we needed to investigate the contribution of APC-mediated downregulation in NXT007. In the present study, we focused on APC-catalyzed inactivation in NXT007-mediated coagulation. A dose-dependent anticoagulant effect of APC in FVIII-deficient plasma spiked with NXT007 was observed using TGA and CWA results (Figure 2A, Supplementary Table S1). Furthermore, dose-dependent APC-catalyzed inactivation in the nonsevere HA model mixed with NXT007 was observed (Supplementary Figure S1), similar to the severe HA model spiked with NXT007 (Figure 2A). Taken together, our results suggest that, as with emicizumab, APC could regulate NXT007-mediated hemostatic reactions through the direct inactivation of FVa despite the higher cofactor activity of NXT007 relative to emicizumab [11].

Previous experiments have demonstrated that the addition of APC to FVIII-deficient plasmas suppresses TG potential [28]. Conversely, several APC inhibitors enhance the coagulation potential in FVIII-deficient plasmas [29,30]. A recent study also demonstrated that blocking the anticoagulant activity of APC improves the coagulation function in HA mice [31]. In the present study, APC-catalyzed inactivation attenuated TGA parameters in FVIII-deficient plasma spiked with NXT007 (the NXT007-mediated condition), similar to that in PNP, and APC was able to inactivate NXT007-mediated coagulation potential even under low FV concentrations. The plasma FV concentration (8-10 μg/mL) was 80-100 times higher than that of FVIII (∼100 ng/mL). In clinical practice, some FV mutations [[32], [33], [34], [35]] are known as APCR, but FVIII mutations showing APCR have not been reported [36]. For these reasons, we speculate that FVa would play an important role relative to FVIIIa in APC-catalyzed inactivation mechanisms and that APC could suppress NXT007-mediated coagulation through FVa inactivation even in the absence of FVIII, similar to emicizumab.

APC inactivates FVIIIa following cleavage at Arg336 and Arg562, and the cleavage at Arg336 exhibits a more dominant role for APC-catalyzed inactivation of FVIIIa than the cleavage at Arg562 [16]. Biochemical studies demonstrated that the specific activities of several single-point FVIII mutants that exhibited 2-5-fold reductions in APC-catalyzed cleavage at Arg336 and Arg562 were similar to wild type [37]. In addition, there has been no report of prothrombotic phenotype with decreased APC-mediated FVIIIa inactivation [36]. Our data indicate that the inhibitory effect of coagulation potential by APC in FVIII-deficient plasmas supplemented with NXT007 was equivalent to that in PNP, supporting the idea that APC-mediated FVIIIa inactivation may not be an indispensable function in regulating hemostatic potential. On the other hand, a recent study reported that an APC-resistant FVIII mutant, Arg336Gln/Arg562Gln (R336Q/R562Q), could enhance hemostatic function in HA mice [36]. The R336Q/R562Q mutant almost completely lost the cleavage at Arg336 and Arg562 [36], indicating that partial resistance to APC-catalyzed FVIIIa inactivation may not influence procoagulant function and that a complete loss of APC-mediated FVIIIa cleavage could enhance coagulation potential.

As opposed to FVIII, several FV point mutations such as R506Q (FV_Leiden_ [32]), R306T (FV_Cambridge_ [33]), and R306G (FV_Hong Kong_ [33]) impair the APC cleavage site, and those such as W1920R (FV_Nara_ [34]) and A2086D (FV_Besançon_ [35]) disturb the APC cleavage reaction, resulting in the association with venous thrombosis. Previous reports suggested that the coagulation function of patients with HA with a FV_Leiden_ mutation was greater than that without a FV_Leiden_ mutation [38]. Also, an earlier case-control study reported that the onset of the first bleeding episode was delayed among children with HA with a FV_Leiden_ mutation [39]. In addition, an *in vitro* study demonstrated that a FV_Leiden_ mutation could improve TG potential in FVIII-deficient plasma [40]. These findings indicate that FV mutations could influence coagulation potential in patients with HA. Our result demonstrated that APC-mediated downregulation was not altered or disturbed by the differences of NXT007 from FVIIIa even under FV_Leiden_ conditions; the result suggests that NXT007 may be applied to patients with HA even under FV mutations, although some dosage adjustment may be required.

Some nonfactor agents, such as emicizumab, concizumab (an anti-TF pathway inhibitor antibody) [41], or fitusiran (an RNA interference therapeutic agent for targeting antithrombin) [42], have been developed. Patients with HA are expected to benefit significantly from these treatment options. However, some thrombotic complications have been reported with the use of these nonfactor replacements. Three TMAs (thrombotic microangiopathy) and 2 venous thromboembolisms (sinus vein thrombosis and superficial thrombophlebitis) under emicizumab prophylaxis were recorded during repeated activated prothrombin complex concentrates treatments [5]. Sinus vein thrombosis was observed in a noninhibitor patient with HA with the combination of fitusiran and a FVIII product [43]. Regarding concizumab, 2 arterial and 3 venous thrombotic adverse events were observed in 3 patients with HA or HB with inhibitors [44]. Considering these reports, we thought it had to be clarified whether NXT007 affects the innate anticoagulant pathways or not. NXT007 has the potential to achieve near-normal levels of coagulation in an HA state while it does not receive degradation by APC differently from FVIIIa. Here, we demonstrated that, even in the NXT007-mediated coagulation, APC-induced inactivation mechanisms worked well in the plasma of patients with HA. Further investigations are ongoing to evaluate the coagulant/anticoagulant balance associated with antithrombin and TF pathway inhibitors in the presence of NXT007.
