# Supplementary figures and images for: NXT007-mediated hemostatic potential is suppressed by activated protein C-catalyzed inactivation of activated factor V
Source: Res Pract Thromb Haemost. 2023 Nov 23;8(1):102271. doi: 10.1016/j.rpth.2023.102271 (PMC10727940; doi:10.1016/j.rpth.2023.102271)

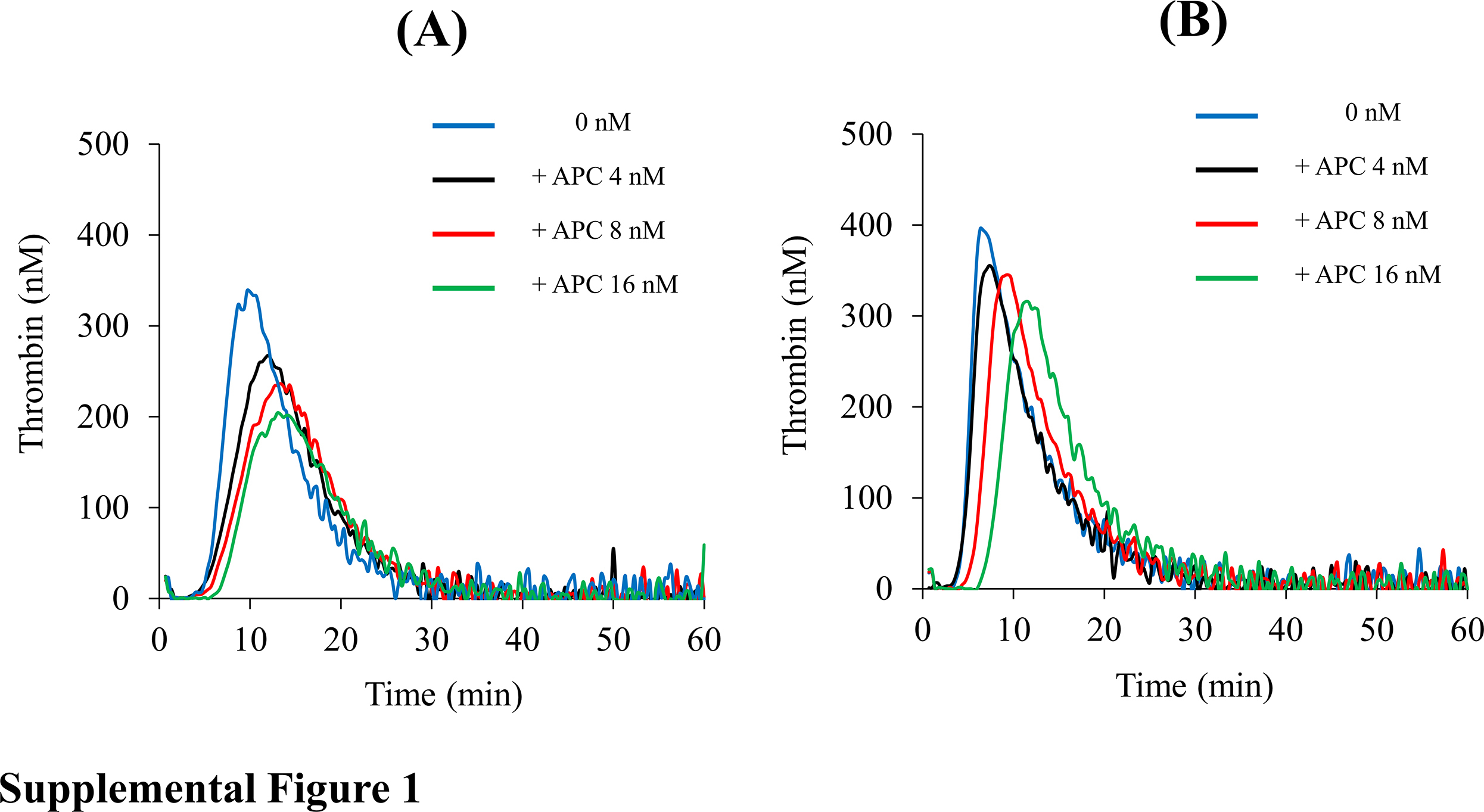

Supplement: Supplementary Figure [file figs1.jpg]
