# Supplementary material for: NXT007-mediated hemostatic potential is suppressed by activated protein C-catalyzed inactivation of activated factor V
Source: Res Pract Thromb Haemost. 2023 Nov 23;8(1):102271. doi: 10.1016/j.rpth.2023.102271 (PMC10727940; doi:10.1016/j.rpth.2023.102271)
Supplement: Supplementary Data [file mmc1.docx]

**Supplemental data**

**Supplementary Figure S1. Effect of APC on FVIII-deficient plasmas spiked with NXT007 together with mild levels of FVIII:C by TGA**

The nonsevere HA model was prepared using FVIII-deficient plasmas and full-length rFVIII (Advate^®^). TF/Elg-triggered thrombin generation after the addition of various APC concentrations (0–16 nM) was examined in FVIII-deficient plasma supplemented with NXT007 (10 µg/mL) and FVIII:C 5 IU/dL (*panel***A**) or 20 IU/dL (*panel***B**). Experiments were performed twice, and representative APC dose-dependent thrombin generation curves are shown (*blue*; no addition, *black*; APC 4 nM, *red*; APC 8 nM, *green*; APC 16 nM).

**Supplementary Table S1.**

**Parameters in normal plasma or FVIII-deficient plasma with NXT007 in the presence of APC by clot waveform assay**

CWA was performed as described in Methods. The obtained parameters in pooled normal plasma (PNP) or FVIII-deficient plasma with NXT007 (10 µg/mL) together with various concentrations of APC are shown. CWA parameters in PNP or FVIII-deficient plasma plus NXT007 with or without APC were compared. Significant differences were considered as p <0.05. Experiments were performed 4 times, and the average values and SDsare shown.

| Parameters | PNP | | | |  | + NXT (10 µg/ml) | | | | Normal  controls |
| --- | --- | --- | --- | --- | --- | --- | --- | --- | --- | --- |
|  | No APC | + APC 4 nM | + APC 8 nM | + APC 16 nM |  | No APC | + APC 4 nM | + APC 8 nM | + APC 16 nM |  |
|  |  |  |  |  |  |  |  |  |  |  |
| Ad\|min1\| | 7.3±0.1 | 6.7±0.2* | 6.1±0.1** | 5.3±0.1** |  | 6.8±0.1 | 6.0±0.1** | 5.5±0.1** | 5.1±0.1** | 7.2±0.6 |
| Clot time (s) | 29±0.2 | 31±0.6* | 34±0.3** | 37±0.8** |  | 28±0.5 | 31±0.4** | 34±0.5** | 36±0.8** | 31±1.6 |

* p <0.05, ** p <0.01*vs*no APC
